# Opposing impacts on healthspan and longevity by limiting dietary selenium in telomere dysfunctional mice

**DOI:** 10.1111/acel.12529

**Published:** 2016-09-21

**Authors:** Ryan T. Wu, Lei Cao, Elliot Mattson, Kenneth W. Witwer, Jay Cao, Huawei Zeng, Xin He, Gerald F. Combs, Wen‐Hsing Cheng

**Affiliations:** ^1^ Department of Nutrition and Food ScienceUniversity of MarylandCollege ParkMD20742USA; ^2^ Department of Food Science, Nutrition and Health PromotionMississippi State UniversityMississippi StateMS39762USA; ^3^ Department of Molecular & Comparative PathobiologyJohns Hopkins UniversityBaltimoreMD21205USA; ^4^ USDAAgricultural Research ServiceGrand Forks Human Nutrition CenterGrand ForksND58202USA; ^5^ Department of Epidemiology and BiostatisticsUniversity of MarylandCollege ParkMD20742USA

**Keywords:** accelerated aging, antioxidant, longevity regulation, mouse models, selenium, trade‐offs

## Abstract

Selenium (Se) is a trace metalloid essential for life, but its nutritional and physiological roles during the aging process remain elusive. While telomere attrition contributes to replicative senescence mainly through persistent DNA damage response, such an aging process is mitigated in mice with inherently long telomeres. Here, weanling third generation telomerase RNA component knockout mice carrying short telomeres were fed a Se‐deficient basal diet or the diet supplemented with 0.15 ppm Se as sodium selenate to be nutritionally sufficient throughout their life. Dietary Se deprivation delayed wound healing and accelerated incidence of osteoporosis, gray hair, alopecia, and cataract, but surprisingly promoted longevity. Plasma microRNA profiling revealed a circulating signature of Se deprivation, and subsequent ontological analyses predicted dominant changes in metabolism. Consistent with this observation, dietary Se deprivation accelerated age‐dependent declines in glucose tolerance, insulin sensitivity, and glucose‐stimulated insulin production in the mice. Moreover, DNA damage and senescence responses were enhanced and *Pdx1* and *MafA* mRNA expression were reduced in pancreas of the Se‐deficient mice. Altogether, these results suggest a novel model of aging with conceptual advances, whereby Se at low levels may be considered a hormetic chemical and decouple healthspan and longevity.

## Introduction

The essential mineral selenium (Se) is involved in a diverse range of chronic diseases primarily through selenoproteins and Se metabolites. Of the 25 known mammalian selenoproteins, 11 are believed to participate in processes of aging (McCann & Ames, 2011). While mice unable to express selenoproteins in epidermal cells (Sengupta *et al*., 2010) or in osteo‐chondroprogenitor cells (Downey *et al*., 2009) show age‐related alopecia and bone abnormality, knockout of glutathione peroxidase‐1, a major selenoprotein accounting for 58% of total Se in liver (Cheng *et al*., 1997), does not render the mice discernible aging phenotypes up to 20 months of age (Ho *et al*., 1997). Although optimal health in mammals requires Se at nutritional levels of intake, cumulative lines of evidence surprisingly point to disease‐promoting roles of certain selenoproteins under distinct conditions (for details, see Lei *et al*., 2016). Furthermore, analyses of liver Se concentrations indicate an inverse association with longevity in 26 species (Ma *et al*., 2015). Despite these advances, how long‐term dietary Se deprivation impacts healthspan and longevity remains ill understood.

Telomere attrition provokes DNA damage response and, subsequently, replicative senescence. Telomeric repeats in human cells are 10–15 kb in length, but the laboratory mouse species *Mus musculus* possesses much longer telomeres, ranging 25–80 kb (Blasco *et al*., 1997; Herrera *et al*., 1999). Furthermore, mice have generally higher levels of somatic telomerase activity than do humans, which may resist telomere attrition. To better model aging in the mouse carrying humanized telomeres, mice with telomerase RNA component (*Terc* ^−/−^) (Blasco *et al*., 1997) and telomerase reverse transcriptase (*Tert* ^−/−^) (Yuan *et al*., 1999) knockouts have been generated. Progressive inbreeding is required to gradually shorten telomeres in *Terc* ^−/−^ mice to a range reminiscent of that in humans. The aging phenotypes of such premature aging syndromes in humans as Werner syndrome and ataxia‐telangiectasia do not appear in mice when only the responsible mutated genes (*Wrn* and *Atm*) have been knocked out. However, those aging phenotypes of *Wrn* ^*−/−*^ and *Atm* ^*−/−*^ mice can be recapitulated under a short telomere background (Wong *et al*., 2003; Chang *et al*., 2004). Here, we used late generation *Terc* ^−/−^ mice as a model to determine the impact of dietary Se deprivation during the aging process. Our results demonstrate that dietary Se deprivation deteriorates healthspan but paradoxically promotes longevity.

## Results

### Dietary Se deprivation increases longevity in G3 *Terc* ^*−/−*^ mice

*Terc* ^−/−^ mice have been generated in the original 60% C57BL/6J, 37.5% 129/Sv and 2.5% SJL mixed (Blasco *et al*., 1997; Gonzalez‐Suarez *et al*., 2003) and then a C57BL/6J background (Herrera *et al*., 1999), whose telomeres average 40 and 25 kb, respectively. Generation 3 (G3) *Terc* ^−/−^ mice under the C57BL/6, 129/Sv and SJL mixed background display skin ulceration, alopecia and hair graying, but many other age‐related disorders, including osteoporosis, body weight changes, shortened lifespan, and type II diabetes, do not appear until G4‐G6 (Rudolph *et al*., 1999). Because we employed a line of *Terc* ^−/−^ mice in a nearly pure C57BL/6J background, such phenotypes were expected at generations earlier than those described by Rudolph *et al*. (1999). Consistent with the observations that infertility occurred at G4 in a pure C57BL/6J and G6 in the mixed background, our *Terc* ^−/−^ mice were fertile until G4. Dietary Se deprivation since weanling induced early onset of hair graying and alopecia at 10 and 7 months of age in G2 and G3 male *Terc* ^−/−^ mice (Fig. S1), respectively, but not in *Terc* ^+/+^ mice (data not shown). Furthermore, dietary Se deprivation had no discernable impact on glucose tolerance in *Terc* ^+/+^ mice at 12 months of age (Fig. S2). Based on these pilot studies, we chose to determine the impact of dietary Se deprivation on age‐related degeneration and longevity only in *Terc* ^−/−^ mice (Fig. S3).

**Figure 1:**
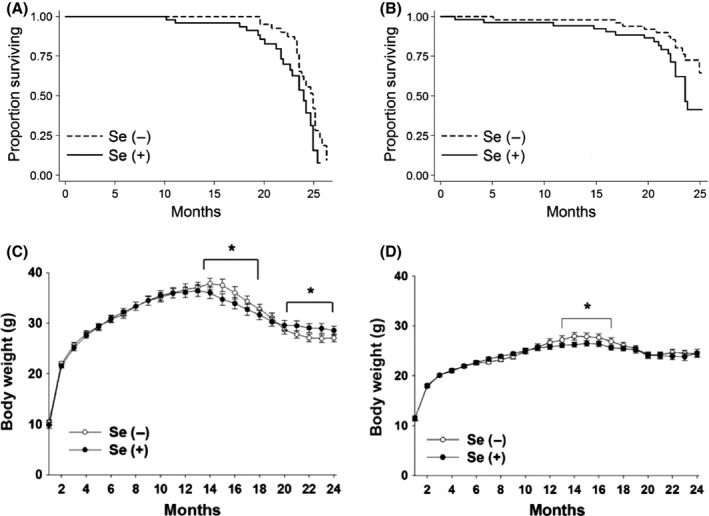
Effect of dietary Se deprivation on survival and body weight in G3 *Terc* ^−/−^ mice. Kaplan–Meier survival curves in males (A) and females (B) were analyzed by log‐rank tests of Se(−) vs. Se(+) G3 *Terc* ^−/−^ mice. Males, *P *=* *0.048 [Se(−), *n* = 52; Se(+), *n* = 49]; females, *P *=* *0.025 [Se(−), *n* = 50; Se(+), *n* = 52]. Lifelong trends of body weights of male (C) and female (D) G3 *Terc* ^−/−^ mice fed a Se(−) or a Se(+) diet. *P <* *0.05, compared to Se(+) mice. Se(−), selenium‐deficient; Se(+), selenium‐adequate.

A total of 203 weanling G3 *Terc* ^−/−^ mice were fed an AIN‐93G Torula yeast Se‐deficient basal diet (Se−, 0.03 ppm by analysis) or the diet supplemented with Se at 0.15 ppm (nutritionally adequate, Se+) as sodium selenate (Holmstrom *et al*., 2012) until they were sacrificed or died of natural causes. Based on the log‐rank test of equality of two survival functions (Peto & Peto, 1972), Se− G3 *Terc* ^−/−^ mice surprisingly exhibited a higher survival probability than Se+ mice over time in both males (*P *=* *0.045, Fig. 1A) and females (*P *=* *0.025, Fig. 1B). Compared to the estimated median survival time of Se+ mice, Se− mice were predicted to live 4 and at least 7 weeks longer in males and females, respectively. We could not precisely determine the ultimate mean lifespan due to mandatory cull of sick mice. Females out‐lived males in both dietary groups. Male and female Se+ G3 *Terc* ^−/−^ mice steadily gained weight until 13 and 16 months of age, respectively, and maximum body weight was greater in males than females. Compared to Se+ mice, body weights in Se− mice increased by 2–8% at 14–18 months in males (Fig. 1C) and 4–7% at 13–17 months in females (Fig. 1D). Interestingly, male Se− G3 *Terc* ^−/−^ mice displayed rapid declines in body weight after 15 months of age such that they were 4–7% lighter than Se+ mice at 20–24 months. Food intake did not differ between Se− and Se+ G3 *Terc* ^*−/−*^ mice in either sex beyond mature age (Fig. S4). Male Se− G3 *Terc* ^−/−^ mice had alterations in plasma markers that implied improved survival, including declined IGF‐1 and triglyceride levels and activity of aspartate aminotransferase at 12 and/or 18 months of age under a fasting or a fed condition (Table S1). These data indicate that dietary Se deprivation promotes longevity and suppresses an aging‐promoting hormone IGF‐1 and metabolic stress in G3 *Terc* ^−/−^ mice.

### Dietary Se deprivation promotes age‐related degenerations in G3 *Terc* ^*−/−*^ mice

**Figure 2:**
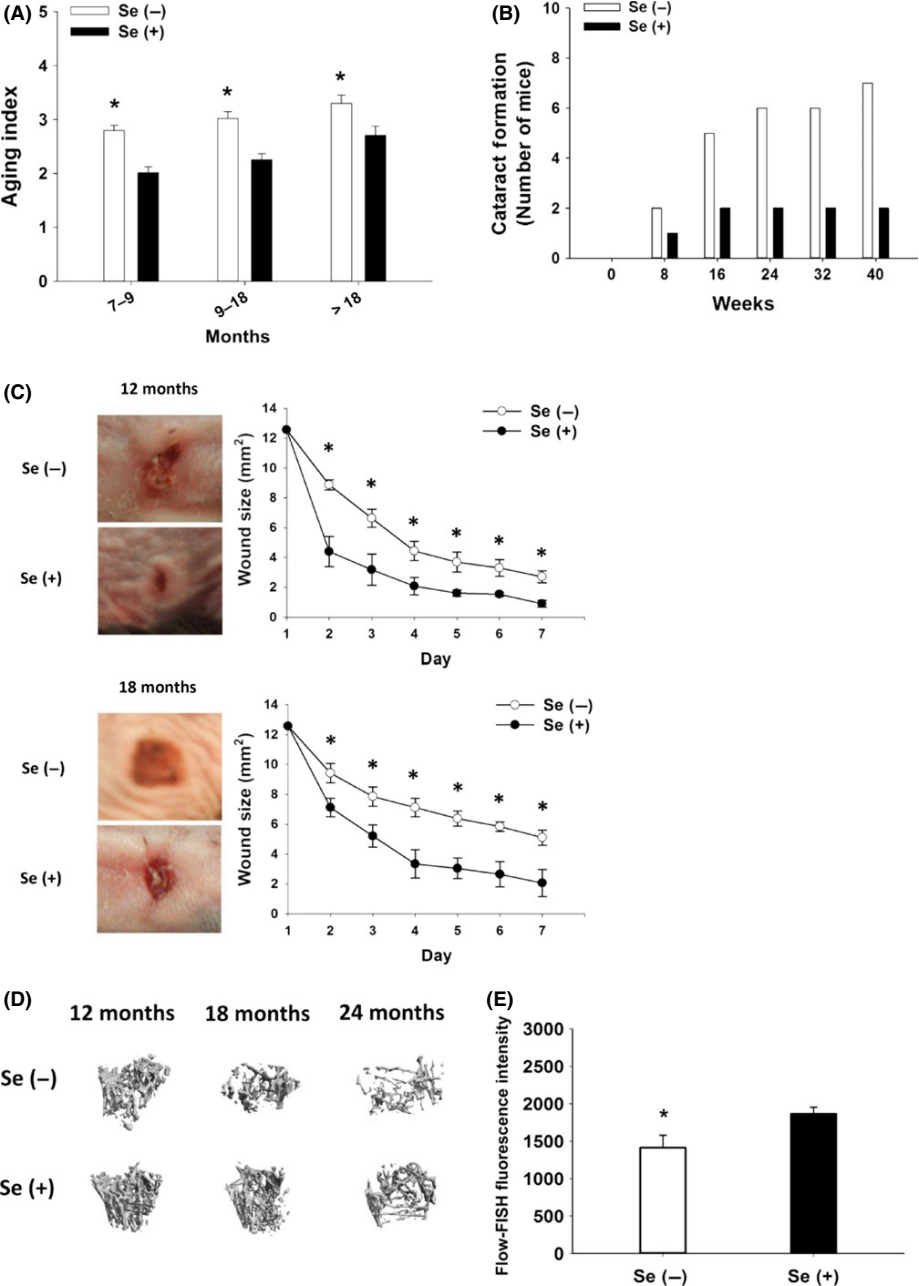
Early onset of aging phenotypes and healthspan deterioration in Se‐deficient male G3 *Terc* ^−/−^ mice. (A) Scores (0, normal; 5, most severe) for hair graying and alopecia. (B) Accumulative incidence of cataract. (C) Wound areas were measured during the 7‐day recovery with a caliper (*n* = 5). Representative pictures of wounds at Day 7 were shown. (D) Representative micro‐computed tomography imaging of re‐constitutive femoral bone structures displaying connectivity density (*n* = 5–8). (E) Flow‐FISH analyses of telomere length using FITC‐telomere‐PNA probe in primary colonocytes isolated from the mice at 24 months of age (*n* = 5). *P <* *0.05, compared to Se(+) mice. Values are means ± SEM. Se(−), selenium‐deficient; Se(+), selenium‐adequate; FISH, fluorescence *in‐situ* hybridization.

Meanwhile, dietary Se deprivation exacerbated hair graying and alopecia (scored 4+; 9–18 months, 32% vs. 9%; > 18 months, 50% vs. 18%; Figs 2A and S5A), cataract formation (Fig. 2B) and spontaneous skin lesion (Fig. S5B), delayed healing of acute wounds on skin (Fig. 2C), and lowered femoral conductivity (Fig. 2D) as the male G3 *Terc* ^−/−^ mice aged. All other parameters of bone health did not differ between the Se− and the Se+ mice (data not shown). Similarly, lactate dehydrogenase activity, an indicator of tissue damage, was increased by aging (84%, *P *<* *0.05) or dietary Se deprivation at 12 months of age (51%, *P *=* *0.069) (Table S1). Results from flow cytometric analysis with fluorescence *in‐situ* hybridization (flow‐FISH) demonstrated telomere shortening by 22% in primary colonocytes isolated from Se− compared to Se+ G3 *Terc* ^−/−^ mice at 24 months of age (Fig. 2E). Dietary Se deprivation reduced plasma Se concentrations by 58% at 12 months and 67% at 18 months of age (Fig. S6A) and levels of plasma glutathione peroxidase‐3, an extracellular selenoprotein and Se marker, by 72% at 12 months and 80% at 18 months of age (Fig. S6B) in G3 *Terc* ^−/−^ mice. While levels of plasma cholesterol, alanine aminotransferase, creatine phosphokinase, alkaline phosphatase, and bilirubin were not affected by dietary Se deprivation or age, amylase activity and levels of albumin and creatine were decreased by aging but not dietary Se deprivation (Table S1). Altogether, dietary Se deprivation in G3 *Terc* ^−/−^ mice promotes age‐related degenerations and telomere shortening in the primary colonocytes.

### Identification of circulating microRNA signature and prediction of metabolic changes in Se‐deficient G3 *Terc* ^*−/−*^ mice

**Figure 3:**
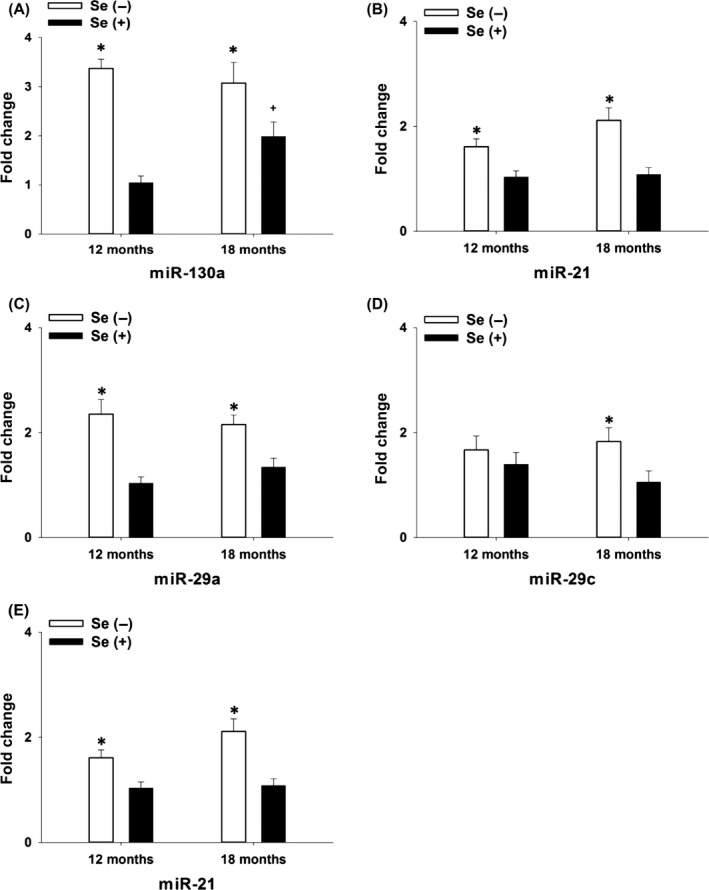
Quantitative RT–PCR analysis of array‐profiled key miRNAs in plasma of Se− and Se+ male G3 *Terc* ^−/−^ mice. Following TaqMan OpenArray profiling, qRT–PCR analyses of plasma miR‐130a, miR‐21, miR‐29a, miR‐29c, and miR‐34a (A–E) were performed. Values are means ± SEM (*n* = 6). *P <* *0.05, compared to Se(+) mice; ^**+**^ *P *<* *0.05, compared to 12 months. Se(−), selenium‐deficient; Se(+), selenium‐adequate.

The molecular mediators linking Se to aging are poorly understood. MicroRNAs (miRNAs) are regulators of mRNA stability and translation and have been proposed as biomarkers for a variety of diseases and physiological conditions including aging. Plasma miRNA profiling is ideal for an assessment of systemic, whole‐body responses as circulating miRNAs are very stable and originate from cells of various origins. Thus, a high‐throughput TaqMan OpenArray platform was employed and the differentially expressed plasma miRNAs were validated using qRT–PCR (Witwer *et al*., 2012). To this end, over 800 miRNAs were profiled in the plasma of male Se− and Se+ G3 *Terc* ^−/−^ mice at 12 and 18 months of age. Of the 131 detectable miRNAs, a couple of them were responsive to dietary Se deprivation, aging, or both (Fig. S7A–C). Individual qRT–PCR assays of top ranked miRNAs, as well as additional ones reported to be aging‐associated (Bilsland *et al*., 2013), demonstrated elevated miR‐130a expression with age in Se+ mice, and dietary Se deprivation exacerbated such changes (Fig. 3A). Similarly, levels of miR‐21, miR‐29a, miR‐29c, and miR‐34a expression were greater in Se− than in Se+ mice, but they were not significantly affected by aging except miR‐34a (*P *=* *0.055) (Fig. 3B–E). Based on the profile of differentially expressed miRNAs, further analyses of the TargetScan/Gene ontology/Panther classification system were performed to predict affected genes and common biological functions. There was a pronounced overlap between the age‐ and Se‐associated miR‐130a and the Se‐associated miRNAs (miR‐21, miR‐29a/c and miR‐34a). Based on the Panther classification system (Mi *et al*., 2013), metabolic process is the most dominant (29–42%) biological functions (Fig. S7D–F). Additionally, the top 10 biological functions of miR‐130a ranked by gene ontology enrichment analysis were also significant targets of miR‐21, miR‐29a/c, and miR‐34a (Fig. S8). Taken together, the plasma miRNA profile predicts metabolism as the most prominent biological function affected by dietary Se deprivation as the G3 *Terc* ^−/−^ mice age.

### Dietary Se deprivation exacerbates age‐dependent metabolic disorders in G3 *Terc* ^*−/−*^ mice

**Figure 4:**
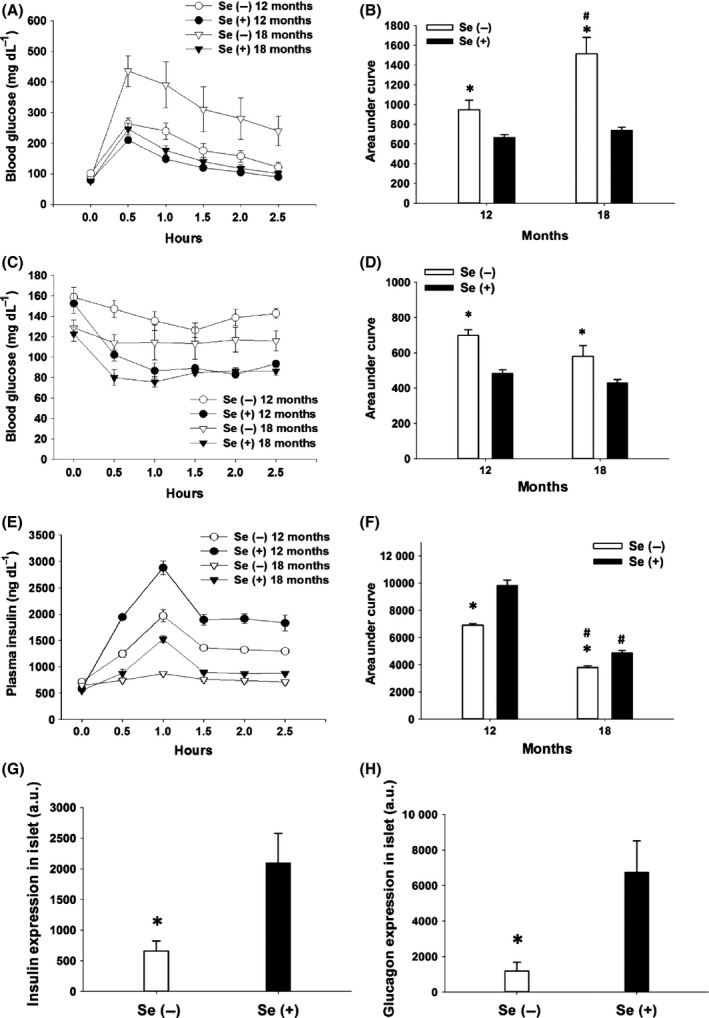
Age‐dependent glucose metabolism defect in Se‐deficient male G3 *Terc* ^−/−^ mice. Levels of blood glucose were measured after an intraperitoneal injection of glucose (1 g kg^−1^, A, B) or insulin (0.25 unit kg^−1^, C, D), and levels of plasma insulin were determined after an intraperitoneal injection of glucose (1 g kg^−1^, E, F) in the mice. Mice were fasted 8 h prior to glucose injection. Average area under curves were calculated based on the results shown in A, C, and E, and one unit is defined as mg h dL ^−1^ (B, D) or ng h dL ^−1^ (F). * *P *<* *0.05, compared to Se(+) mice; ^**#**^ *P *<* *0.05, compared to 12 months. Quantification of insulin (G) and glucagon (H) expression in the islets of the mice at 24 months of age was shown. a.u., arbitrary unit. Values are means ± SEM (*n* = 6). Se(−), selenium‐deficient; Se(+), selenium‐adequate.

Because ontological analyses of the plasma miRNA profile predicted Se regulation on metabolic modulations as the top pathway (Fig. S7D–F) and fasting plasma glucose in Se− mice were elevated (Table S1), we next investigated the physiological impact of dietary Se deprivation on glucose metabolism in male G3 *Terc* ^−/−^ mice at 12 and 18 months of age. There were glucose intolerance (Fig. 4A,B) and insulin resistance in Se− mice (Fig. 4C,D). To determine whether the glucose intolerance phenotype is associated with insulin level, glucose‐stimulated insulin secretion tests were performed and the results demonstrated declines in plasma insulin levels in Se− mice to the extent that there was almost no detectable insulin after glucose injection at 18 months of age (Fig. 4E,F). In Se+ mice, aging deteriorated glucose‐induced insulin secretion, but not glucose tolerance or insulin sensitivity. Further immunohistochemical analyses of sectioned pancreas (Fig. S9A) demonstrated that dietary Se deprivation reduced insulin (Fig. 4G) and glucagon (Fig. 4H) expression by 68% and 82%, respectively, in G3 *Terc* ^−/−^ mice at 24 months of age; however, cell density in pancreatic islets did not significantly differ between the Se− and Se+ mice (Fig. S9B). Altogether, these results strongly implicate dietary Se deprivation in both insulin resistance and defective insulin production under pathophysiological conditions reminiscent of late stage type‐2 diabetes as the G3 *Terc* ^−/−^ mice age.

### Dietary Se deprivation down‐regulates insulin‐related genes in pancreas of G3 *Terc* ^*−/−*^ mice

**Figure 5:**
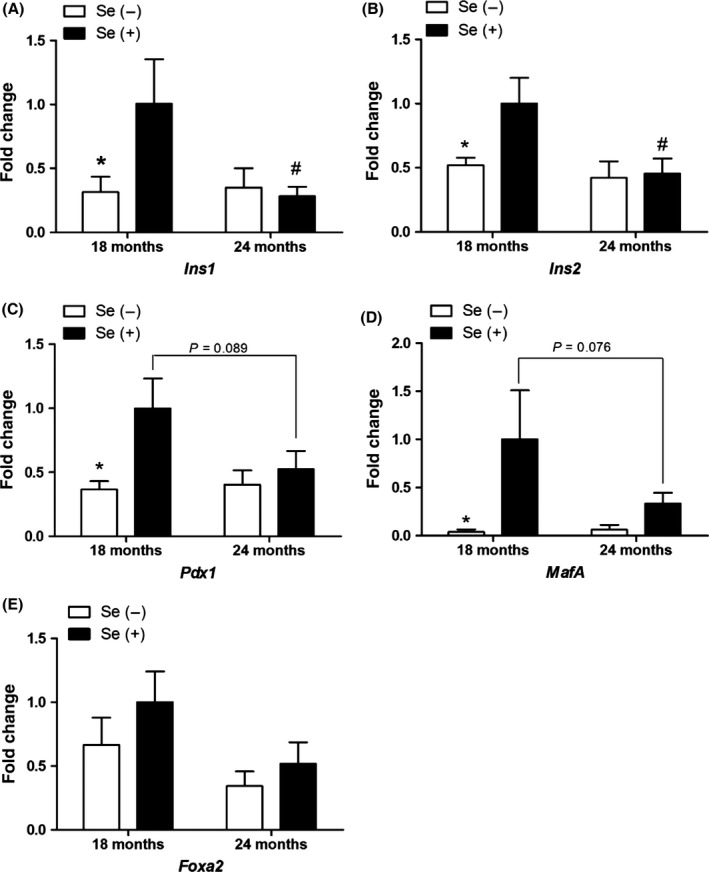
Early onset of age‐dependent declines in key mRNAs for insulin synthesis and secretion in pancreas of Se‐deficient male G3 *Terc* ^−/−^ mice. Quantitative RT–PCR analyses of pancreatic *Ins1*,* Ins2*,* Pdx1*,* MafA*, and *Foxa2* mRNA expression (A–E) were performed in the mice. Values are means ± SEM (*n* = 6–9). *P <* *0.05, compared to Se(+) mice; ^#^ *P *<* *0.05, compared to 18‐month. Se(−), selenium‐deficient; Se(+), selenium‐adequate.

To gain further insight into reduced glucose‐stimulated insulin secretion by dietary Se deprivation, qRT–PCR analyses were performed to determine insulin‐related mRNA expression in the pancreas. The expression of insulin mRNAs declined as the mice aged, but mice on a Se− diet exhibited an early onset of insulin insufficiency (Fig. 5A,B). Pdx1, MafA, and Foxa2 are critical transcriptional factors for insulin synthesis and secretion in pancreatic β‐cells. The mRNA expression of *Pdx1* and *MafA*, but not *Foxa2*, was marginally decreased by aging but greatly reduced by dietary Se deprivation at 18 months of age (Fig. 5C–E). Therefore, the observed insulin insufficiency in Se− G3 *Terc* ^−/−^ mice appears to be linked to *Pdx1* and *MafA* under‐expression.

### Dietary Se deprivation exacerbates senescence induction and genome instability in pancreas of G3 *Terc* ^*−/−*^ mice

**Figure 6:**
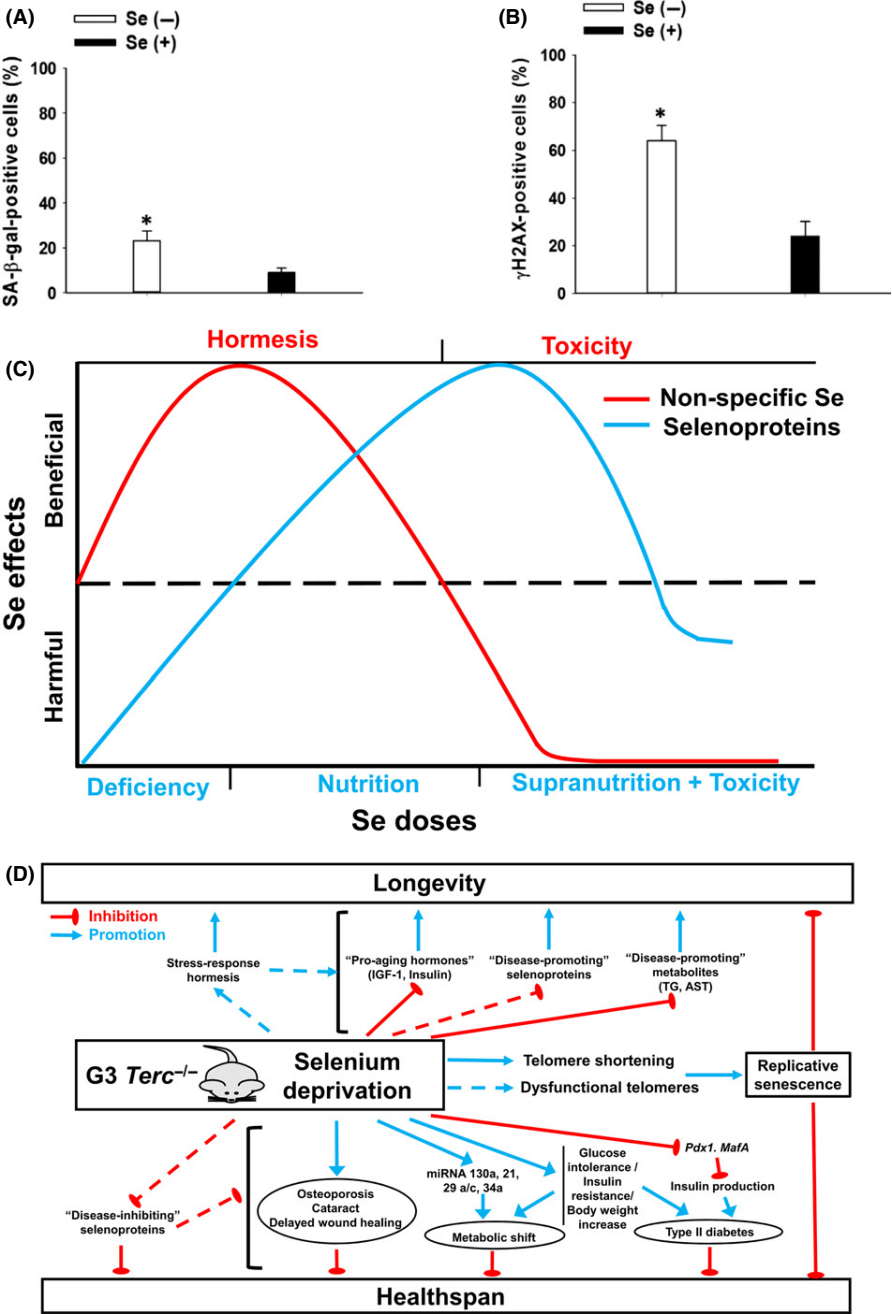
Exacerbated induction of senescence and γH2AX in pancreas of old Se‐deficient male G3 *Terc* ^−/−^ mice and illustrations for the proposed paradoxical role of Se in longevity and healthspan. Senescence‐associated β‐galactosidase expression (A) and immunohistochemical analysis of γH2AX expression (B) in pancreas of the mice at 24 months of age were performed. Values are means ± SEM (*n* = 6–9). *P <* *0.05, compared to Se(+) mice. Se(−), selenium‐deficient; Se(+), selenium‐adequate. (C) An illustration for the Se dose‐dependent effect on optimal health through nonspecific Se at nutritionally deficient levels that elicits hormetic responses or selenoproteins at nutritional levels that modulate physiological functions. (D) The effect of dietary Se deprivation on longevity and healthspan in G3 *Terc* ^−/−^ mice. Phenotypes or pathways affected by dietary Se deprivation and their impacts on increased longevity and weakened healthspan are shown. Broken lines denote those that are supported by literatures but require experimental verification in the mice. These two illustrations are elaborated in the discussion section.

Early onset of senescence and genome instability are hallmarks of *Terc* ^−/−^ mice (Chang *et al*., 2004). We found that dietary Se deprivation significantly increased the expression of senescence‐associated β‐galactosidase (Figs 6A and S9C) and γH2AX (Figs 6B and S9D) in pancreas of G3 *Terc* ^−/−^ mice at 24 months of age. Further analyses of genes in association with senescence and DNA damage response demonstrated lower levels of *Lmnb1* mRNA at 24 than 18 months of age in pancreas of G3 Se+ *Terc* ^−/−^ mice, and dietary Se deprivation exacerbated such declines at 18 months of age (Fig. S10A). Although activation of the p53‐p21 pathway was known to be linked to ROS and DNA damage‐induced senescence (Munoz‐Espin & Serrano, 2014), this event seemed to be dispensable in the senescent *Terc* ^*−/−*^ pancreas as the mRNA levels of *p21* (Fig. S10B) and *Tp53* (Fig. S10C) were decreased and unaffected, respectively, by aging or dietary Se deprivation. These results collectively suggest that dietary Se deprivation promotes senescence and DNA damage in pancreas of G3 *Terc* ^−/−^ mice.

## Discussion

Although healthspan is usually positively associated with longevity, here we report a paradoxical effect of dietary Se deprivation on longevity promotion together with the expected healthspan deterioration observations in short telomere mice. As illustrated in Fig. 6C, we propose that such intriguing results may be attributed to a dual role of Se in physiological maintenance in the form of selenoproteins at nutritional levels of intake and hormesis through forms other than selenoproteins at low or nutritionally deficient levels. In this regard, dietary intake of Se at given dosage exhibits both beneficial and harmful effects that define healthspan and lifespan through selenoproteins and nonspecific Se pool, depending of individual selenoproteins and doses of Se. Selenoproteins, nonselenoproteins and low molecular weight species are estimated to account for 52%, 46% and 2% of total Se, respectively, in plasma of healthy humans (Combs *et al*., 2012). Selenoproteins recode the stop codon UGA for selenocysteine incorporation, but such codon duality put selenoprotein expression at risk of truncation mutations and degradation. While expression of certain selenoproteins under Se deficiency is prematurely terminated by nonsense‐mediated decay of selenoprotein mRNA and a CRL2 ubiquitin ligase‐mediated degradation (Seyedali & Berry, 2014; Lin *et al*., 2015), these two pathways are less likely to degrade nonselenoproteins. Thus, Se in principle may be present to a proportionally increased extent in the form of hormetic Se in Se‐deficient mice. It is plausible that, under Se deficiency, decreased expression of selenoproteins overall contributes to the phenotypes of healthspan deterioration despite of under‐expression of certain disease‐promoting selenoproteins (Fig. 6D, elaborated below), but Se in the nonspecific pool may be better retained and acts as a hormetic chemical for lifespan extension. To the best of our knowledge, this is the first report that a dietary condition decouples healthspan and longevity.

An apparent and puzzling question is: Why do Se‐restricted mice with declining healthspan in every measurement show increased longevity? As illustrated (Fig. 6C), it is tempting to conjecture that longevity benefits offered by hormetic Se at low levels counteract disadvantages from selenoprotein deficiency, resulting in a positive net value. Such a sum is greater in deficient than nutritional levels of Se, propitiously explaining the observed longevity promotion by dietary Se deprivation. Although this model is highly speculative, it is favored considering the following three lines of evidence.

First, declined IGF‐1 and insulin protein levels in the plasma of Se‐deficient mice may be a prominent determinant (Fig. 6D). There is evidence that disrupted IGF‐1/insulin signaling in *Drosophila* and mice extends lifespan, but promotes metabolic dysregulation and age‐related disorders (Rincon *et al*., 2004; Broughton *et al*., 2005). We observe that fasting IGF‐1 levels are declined by dietary Se deprivation at 12 but not 18 months of age, which is in line with results of the analyses of 30 strains of mice by the Jackson Laboratory that show an inverse association of lifespan with IGF‐1 levels only early in life (Yuan *et al*., 2009). Furthermore, although defective glucose‐stimulated insulin production by dietary Se deprivation (Fig. 4E) exacerbates glucose intolerance and diabetes in the mice, such a condition may oppositely promotes longevity through reduced metabolism (Zhang & Liu, 2014). Nonetheless, inducible knockout of liver‐specific *Igf1* in mice starting at 12 months of age results in reduced serum IGF‐1 levels by 70% and impaired healthspan (Gong *et al*., 2014). It is of future interest to elucidate whether Se regulation of IGF‐1/insulin signaling on lifespan and healthspan is age‐dependent.

Second, stress‐induced hormetic responses may favor longevity as a consequence of dietary Se deprivation (Fig. 6C). A substantial decline in body Se status can be achieved as early as 5 weeks in mice on a Se‐deficient diet, and dietary Se requirement in 1‐year old rats is dropped by 50% (Sunde & Thompson, 2009). In our 1‐year old mice on a Se‐deficient diet since weanling, plasma Se concentration and Gpx3 protein level is decreased by 58% and 72%, respectively, suggesting that the remaining Se is better retained in the nonspecific pool than selenoproteins. In Se‐deficient mice, hormetic Se and decreased expression of antioxidant selenoproteins may collectively induce chronic and modest oxidative stress, resulting in longevity promotion (Ristow & Schmeisser, 2011). Of note, Se‐dependent antioxidant enzymes only represent a small fraction of total antioxidants (Lei *et al*., 2016). Considering Se as a hormetic chemical, Se‐deficient mice may be exposed to chronic oxidative stress early in life and adapt to such a pro‐longevity, mildly stressed condition. Consistent with this hypothesis, the Gladyshev Lab has recently shown an inverse association between liver Se content and longevity across 26 mammalian species (Ma *et al*., 2015). Interestingly, 50% reduction of *Gpx4* expression in mice results in a slight (7%) yet statistically significant lifespan extension, although they accumulate oxidized lipids in the brain (Ran *et al*., 2007). Thus, it is explicable yet provocative to propose that Se at very low levels well below nutritional needs may promote longevity through stress‐response hormesis.

Third, depending on the physiological context, some of the 24 mouse selenoproteins can promote disease such that down‐regulation of them may promote healthspan and thus contribute to longevity. For example, expression of a couple of selenoproteins, including Gpx1, selenoprotein P and selenoprotein M, is associated with insulin resistance and/or obesity. Furthermore, *Sep15* ^−/−^ mice display less chemically induced aberrant crypt foci than controls, and liver‐specific *Txnrd1* ^−/−^ mice are resistant to APAP‐induced hepatotoxicity (Lei *et al*., 2016). While awaiting experimental verification, Se at nutritional levels of intake that supports full selenoprotein expression is likely a double‐edged sword as it promotes healthy aging through ‘disease‐inhibiting’ selenoproteins but deteriorates healthspan through ‘disease‐promoting’ selenoproteins (Fig. 6D).

Mouse models are useful to test theories of aging, but one caveat is that they display limited replicative senescence due to relatively long telomeres. This may explain why current models of dietary Se or selenoprotein deficiency in mice do not recapitulate normal aging sufficiently or comprehensively. Although *Gpx1* ^−/−^ mice do not show aging phenotypes up to 20 months of age (Ho *et al*., 1997), primary *Gpx1* ^−/−^ embryonic fibroblasts (de Haan *et al*., 2004) and selenoprotein H knockdown MRC‐5 cells (Wu *et al*., 2014) show senescence‐like features in a ROS‐dependent manner. Furthermore, heterozygous mutations in *SECISBP2*, a gene encoding SBP2 essential for the expression of all selenoproteins, lead to shortened telomeres in humans (Schoenmakers *et al*., 2010), and such effect appears to be independent of telomerase (Squires *et al*., 2009). The linkage between selenoprotein deficiency and telomere shortening or senescence may be attributed to increased oxidative stress, as many selenoproteins are oxidoreductases. Consistent with this notion, dietary Se deprivation results in telomere shortening in the highly proliferative colonocytes derived from G3 *Terc* ^−/−^ mice at an advanced age (Fig. 2E) and accelerates the onset of skin aging by three months in G3 compared to G2 *Terc* ^−/−^ mice (Fig. S1). Critically short or dysfunctional telomeres in pancreas of Se‐deficient mice may promote pancreatic dysfunction, as they display high levels of DNA damage and senescence. Perhaps as a feedback regulation, persistent DNA damage response may systemically disrupt IGF‐1 signaling that induces metabolic shift minimizing further damage and is pro‐longevity, as observed in the *Ercc1* ^−/−^ progeroid mice (Niedernhofer *et al*., 2006). Altogether, we have provided the first physiological evidence in mice carrying humanized telomeres that dietary Se deficiency induces telomere shortening and senescence.

In humans, aging is associated with numerous pathological conditions including elevated blood glucose and body weight. Roles of Se and selenoproteins in glucose metabolism are complicated. While mice consuming Se above nutritional needs display insulin resistance, expressing an i6A^−^ mutant selenocysteine tRNA for reduced expression of multiple selenoproteins renders the mice glucose intolerance and diabetes‐like symptoms (Labunskyy *et al*., 2011). Moreover, Gpx1 is necessary to restore nuclear MafA localization for optimal insulin expression in pancreatic β‐cells of *db/db* obese mice (Guo *et al*., 2013). By contrast, results from mice engineered to be *Gpx1* null or overexpression with 28‐fold activity increase in the pancreas collectively implicate Gpx1 in diabetes promotion (Wang *et al*., 2008; Loh *et al*., 2009). Furthermore, knockout of selenoprotein P, an extracellular peroxidase, renders the mice improved glucose tolerance and enhanced insulin signaling in the liver and the muscle (Misu *et al*., 2010). Thus, both oxidative and reductive stress may compromise oxygen tone and, subsequently, result in defective insulin signaling. This leads us to propose that there are peroxidase‐independent selenoproteins that account for the Se protection against diabetes at nutritional levels of intake. Considering the transactivation functions of MafA, Pdx1 and Foxa2, it appears that the early onset of insulin decline in the Se‐deficient short telomere mice may be associated with defective glucose‐stimulated insulin secretion but not pancreatic β cell maturation. Although challenging, it is of physiological significance to pinpoint specific selenoproteins that are positive or negative regulators of glucose metabolism and determine their appropriate expression levels for optimal healthspan.

Aging is a biological process characterized by a progressive and accumulative impairment of the organism's response to exogenous and endogenous stress. Results from our studies suggest that dietary Se deprivation oppositely impacts longevity and healthspan through distinct metabolites and molecular markers in G3 *Terc* ^−/−^ mice. Se as an essential mineral at nutritional levels of intake protects against physiological deterioration through certain selenoproteins, but Se as a chemical at nutritionally deficient or very low levels may contribute to longevity by stress‐response hormesis. This raises an interesting scenario and a dilemma, at least for nutrients or bioactive compounds with dual or paradoxical functions, in that diets do not always impact healthy aging and longevity unilaterally. It is tempting to propose that Se mediates healthspan and lifespan mainly through its dual roles in the forms of selenoproteins or a hormetic chemical. The actual impact of Se at a given dose on longevity is additively determined by the harmful and beneficial effects attributed to both selenoproteins and hormetic Se (Fig. 6C). If one considers Se from a toxicity perspective, the Se dosage (0.03 ppm by analysis) in the Se‐deficient basal diet is likely to be a hormetic dose that promotes longevity. Such longevity‐promoting feature of Se at low doses may be compromised or diluted by increased expression of selenoproteins in mice on a Se+ diet (0.15 ppm Se). In the G3 *Terc* ^−/−^ mice, a given dose of Se may not concurrently support both selenoprotein and hormetic Se for optimal health and longevity. Considering that the 25 human selenoproteins distinctly promote or protect different chronic diseases under various stages of pathogenesis and life cycle (Lei *et al*., 2016), the emerging task is to identify intervention strategies that target specific selenoproteins to maximize benefit for both healthspan and lifespan. Another interesting line of future research is to confirm such a paradoxical role of Se in other mouse models of telomere dysfunction or age‐related degeneration (Wong *et al*., 2003; Chang *et al*., 2004), long telomere mice, and species that do not exhibit telomere attrition.

## Experimental procedures

### Mice and diet

*Terc* ^+/−^ mice were backcrossed to be > 99% pure C57BL/6 background (#004132, the Jackson Laboratory) and were bred 3–4 generations before being interbred for the production of *Terc* ^*−/−*^ mice for this study (Fig. S3). Cousins or second cousins of G2 *Terc* ^*−/−*^ mice were bred, and the G3 littermates were equally and randomly assigned to the experimental groups on a Se− or a Se+ diet (Fig. S3). Mice were kept under aseptic conditions in individually ventilated cages (up to four mice/cage) within an animal room (22 °C, 12‐h dark:light cycle) and had *ad libitum* access to foods and sterilized water. The AIN‐93G purified diets (Dyets Inc., Bethlehem, PA, USA) were stored at −20 °C until being consumed. Our diets for adult mice are based on AIN‐93G instead of AIN‐93M because the former includes greater amount of fat and protein that better represents the dietary components of Western foods. The basal (Se−) diet contained 30% Torula yeast and 0.03 mg kg^−1^ Se by analysis (AOAC Method 986.15; Covance Laboratory, Madison, WI, USA) and was supplemented with Se at 0.15 mg kg^−1^ (Se+) as sodium selenate (Holmstrom *et al*., 2012). Selenate is the form of Se in standard AIN‐93 diets and is considered to be more bioavailable and less toxic than selenite. Weanling mice were fed the Se− or Se+ diet until they were sacrificed or died naturally.

### Mouse growth, survival analysis, and age‐related degenerations

Mice were weighed weekly, monitored closely for signs of malnutrition, bullying and ill health, and were separated or killed if they were profoundly ill as determined by in‐house veterinarians and a score system (Table S4) approved by the IACUC of the University of Maryland at College Park. Mouse deaths were scored and analyzed by Kaplan–Meier analysis and a log‐rank test (Peto & Peto, 1972). Femurs with adherent tissues cleaned were analyzed by microcomputated tomography to determine pathological changes. Wound healing capability was temporally assessed after carrying out a 5‐mm diameter, full thickness biopsy to the panniculus carnosus using a dermal skin punch (Chang *et al*., 2004). Graying of the hair, skin lesion and cataract‐like conditions were determined as described previously (Rudolph *et al*., 1999). Because dietary Se is absorbed in intestines and age‐dependent telomere attrition is known to be more prominent in colonocytes than leukocytes in ulcerative colitis patients (Risques *et al*., 2008), colonocytes were isolated (Abolhassani *et al*., 2008) for estimation of telomere length by flow‐FISH with a FITC‐conjugated (CCCTAA)_3_ PNA probe (0.3 μg ml^−1^; Bio‐Synthesis Inc., Lewisville, TX, USA) as previously described (Wang *et al*., 2010).

### Plasma markers and assays for glucose tolerance, insulin sensitivity, and glucose‐induced insulin secretion

Except insulin and IGF‐1 whose levels were determined using an ELISA kit (80‐INSMS‐E01, 22‐IG1MS‐E01; ALPCO Diagnostics, Salem, NH, USA), other plasma markers (Table S1) were measured by AniLytics Inc. (Gaithersburg, MD, USA). Whole blood glucose level was determined using the blood from mouse tails and by the Bayer Contour system (Bayer, Elkhart, IN, USA). For glucose tolerance and glucose‐induced insulin secretion assays, mice being fasted 8 h were i.p. injected with glucose (1 g kg body weight^−1^). For insulin sensitivity test, nonfasting mice were i.p. injected with insulin (0.25 unit kg body weight^−1^; Sigma‐Aldrich, St. Louis, MO, USA). Area under curve analyses (Stephens *et al*., 2012) and plasma Se concentrations (Holmstrom *et al*., 2012) were determined as previously described. Plasma Gpx3 protein was analyzed by Western analysis using 0.3 μL plasma and anti‐Gpx3 (1:2000) and anti‐albumin (1:2000) antibodies (R&D Systems, Minneapolis, MN, USA).

### Immunohistochemistry and senescence assay

Frozen tissues were sectioned and slides prepared by Histoserv, Inc. (Germantwon, MD, USA). Immunohistochemisty of γH2AX was performed following the instruction of the anti‐rabbit HRP‐DAB Cell & Tissue Staining Kit (R&D systems) with an anti‐γH2AX antibody (1:100; Sigma‐Aldrich). For insulin and glucagon staining, slides were fixed in 4% paraformaldehyde, permeabilized in 0.2% Triton X‐100 in PBS, blocked in a solution containing 5% normal donkey serum, 1% bovine serum albumin, 0.2%, gelatin and 0.2% Triton X‐100 in PBS, hybridized with anti‐insulin and anti‐glucagon antibodies together (1:500; Abcam, Cambridge, MA, USA) for 16 h at 4 °C, washed twice in PBS with 0.2% Triton X‐100 for 30 min and once in PBS for 30 min, hybridized with secondary antibodies (1:200, anti‐mouse Alexa Fluor 488 and anti‐rabbit Alexa Fluor 568; Invitrogen, Carlsbad, CA, USA), and then washed as described above. Finally, the slides were mounted with ProLong Gold Antifade reagent containing DAPI (Invitrogen). The expression of senescence‐associated β‐galactosidase was determined following the instruction of a Senescence Detection Kit (BioVision, San Francisco, CA, USA). Images were visualized and captured under a Nikon Eclipse E600 fluorescence microscope (Nikon Inc., Melville, NY, USA).

### miRNA analyses of plasma samples

Sodium citrate‐containing plasma (100 μL) was used for total RNA isolation using the mirVana protocol for liquid samples (Ambion, Austin, TX, USA). The spiked‐in synthetic cel‐miR‐39 was added in the lysis/denaturant buffer before mixing with plasma samples. Procedures for miRNA profiling by the TaqMan OpenArray system (Applied Biosystems, Foster City, CA, USA) were detailed in Fig. S7. Differentially expressed miRNAs were verified by qRT–PCR. The reverse transcription step specifically amplified mature miRNAs by inventoried stem‐loop primers from Applied Biosystems, after which samples were diluted in RNase‐free water (30 μL). Diluted cDNA (2 μL) was combined with a 10 μL solution containing PCR reagents (Applied Biosystems), primers, and probes, and amplified for 45 cycles with an iQ5 Real‐Time PCR machine (Bio‐Rad, Hercules, CA, USA). Delta‐delta threshold cycle (ddCt) method included normalization to cel‐miR‐39 spike‐in.

### qRT–PCR analyses of pancreatic samples

Tissues were stored in TRIzol prior to RNA extraction using chloroform and isopropanol (Fisher Scientific, Fair Lawn, NJ, USA). Reverse transcription was performed following instructions of the High‐Capacity cDNA Reverse Transcription Kit (Invitrogen). Then, cDNA was reacted with Universal SYBR Green Supermix (Bio‐Rad) and specific primer pairs (Table S3) for qPCR analyses using the ABI 7500 Fast Real‐Time PCR System (Life Technology, Carlsbad, CA, USA).

### Statistics

Two‐way factorial analysis of variance (ANOVA) followed by Tukey's *post hoc* tests or single factor ANOVA followed by Student's *t*‐test were used for comparisons of the means by SigmaPlot 12 (Systat Software Inc., San Jose, CA, USA). Survival analysis was conducted using Stata 13 (StataCorp. LP, College Station, TX, USA).

### Study approval

Our experiments were approved by the IACUC Committee of University of Maryland and were conducted in accordance with the NIH guidelines for the care and use of experimental animals.

## Author contributions

R.T.W. and W.H.C. conceived the study and designed the experiments. R.T.W. performed all experiments except indicated otherwise. L.C. performed pancreatic qRT–PCR and plasma Gpx3 analyses. E.M. assisted with animal management and biochemical assays. K.W.W. performed some TaqMan profiling analyses. J.C., H.Z, and G.F.C. performed Se and bone analyses. X.H. performed survival analysis and provided statistical advice. R.T.W and W.H.C. wrote the manuscript, with contributions from other co‐authors.

## Conflict of interest

The authors declare that they have no conflict of interest.

## Funding

This work was supported by the US Department of Agriculture, Agricultural Research Service, CRIS projects 5450‐51000‐050‐00D (to H.Z.) and 3062‐51000‐053‐00D (to J.C.), seed funding from the Department of Molecular and Comparative Pathobiology, the Johns Hopkins University (to K. W.W.), and Maryland Agricultural Experiment Station and Mississippi Agricultural and Forestry Experimental Station (to W.H.C.).

## Supporting information


**Fig. S1** Onset of the skin aging phenotypes induced by dietary Se deprivation is three months earlier in male G3 than in G2 *Terc* ^−/−^ mice.
**Fig. S2** Glucose tolerance test in male *Terc* ^+/+^ mice at 12 months of age.
**Fig. S3** Breeding scheme for the generation of short telomere mice.
**Fig. S4** Average food intake in G3 *Terc* ^−/−^ mice.
**Fig. S5** Skin aging in the male G3 *Terc* ^*−/−*^ mice.
**Fig. S6** Se concentrations and glutathione peroxidase‐3 protein levels in plasma of male G3 *Terc* ^−/−^ mice.
**Fig. S7** miRNA OpenArray analyses of plasma samples and the subsequent ontological analyses in male G3 *Terc* ^*−/−*^ mice.
**Fig. S8** The top 10 biological pathways targeted by miR‐130a, miR‐21, miR‐29a/c, and miR‐34a.
**Fig. S9** Representative pictures and quantification of cell density in pancreas of male G3 *Terc* ^−/−^ mice.
**Fig. S10** Effect of dietary Se deprivation and aging on mRNA expression of three senescence‐related genes in pancreas of male G3 *Terc* ^−/−^ mice.
**Table S1** Biomarkers in plasma of Se‐deficient and Se‐adequate male G3 *Terc* ^−/−^ mice.
**Table S2** Definition of the key pathways named in the Panther classification system.
**Table S3** Primers used for qRT‐PCR analyses of pancreatic mRNA expression.
**Table S4** The pain score sheet used in the study to monitor general health and behavior of the mice.
