# Supplementary material for: Opposing impacts on healthspan and longevity by limiting dietary selenium in telomere dysfunctional mice
Source: Aging Cell. 2016 Sep 21;16(1):125–35. doi: 10.1111/acel.12529 (PMC5242309; doi:10.1111/acel.12529)
Supplement: Supplementary file 1 — Fig. S1 Onset of the skin aging phenotypes induced by dietary Se deprivation is three months earlier in male G3 than in G2 Terc −/− mice. Fig. S2 Glucose tolerance test in male Terc +/+ mice at 12 months of age. Fig. S3 Breeding scheme for the generation of short telomere mice. Fig. S4 Average food intake in G3 Terc −/− mice. Fig. S5 Skin aging in the male G3 Terc −/− mice. Fig. S6 Se concentrations and glutathione peroxidase‐3 protein levels in plasma of male G3 Terc −/− mice. Fig. S7 miRNA OpenArray analyses of plasma samples and the subsequent ontological analyses in male G3 Terc −/− mice. Fig. S8 The top 10 biological pathways targeted by miR‐130a, miR‐21, miR‐29a/c, and miR‐34a. Fig. S9 Representative pictures and quantification of cell density in pancreas of male G3 Terc −/− mice. Fig. S10 Effect of dietary Se deprivation and aging on mRNA expression of three senescence‐related genes in pancreas of male G3 Terc −/− mice. Table S1 Biomarkers in plasma of Se‐deficient and Se‐adequate male G3 Terc −/− mice. Table S2 Definition of the key pathways named in the Panther classification system. Table S3 Primers used for qRT‐PCR analyses of pancreatic mRNA expression. Table S4 The pain score sheet used in the study to monitor general health and behavior of the mice. [file ACEL-16-125-s001.pdf]

SUPPLEMENTAL INFORMATION

Supplemental information includes 10 figures and 4 tables

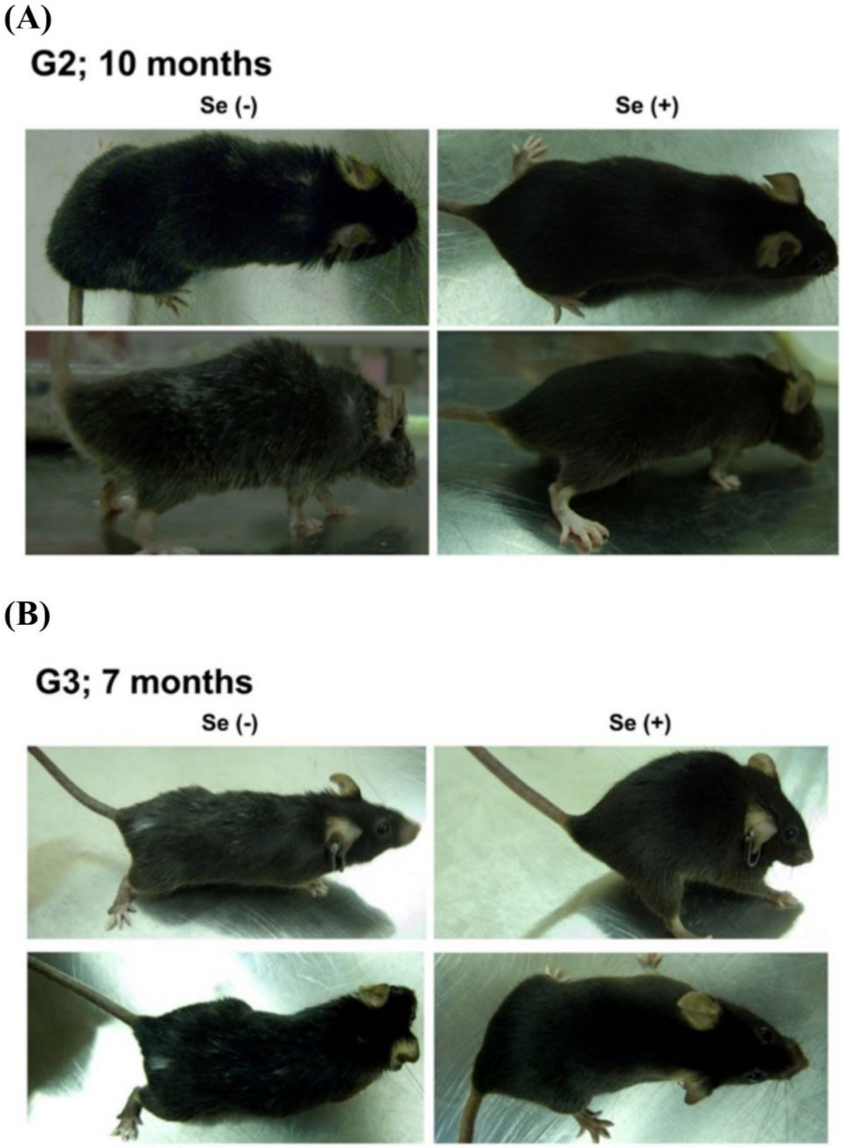

Figure S1. Onset of the skin aging phenotypes induced by dietary Se deprivation is 3 months earlier in male G3 than in G2 *Terc*$^{-/-}$ mice. Representative pictures were taken at 10 and 7 months of age from G2 (**A**) and G3 *Terc*$^{-/-}$ mice (**B**), respectively. Se(**-**), selenium-deficient; Se(+), selenium-adequate.

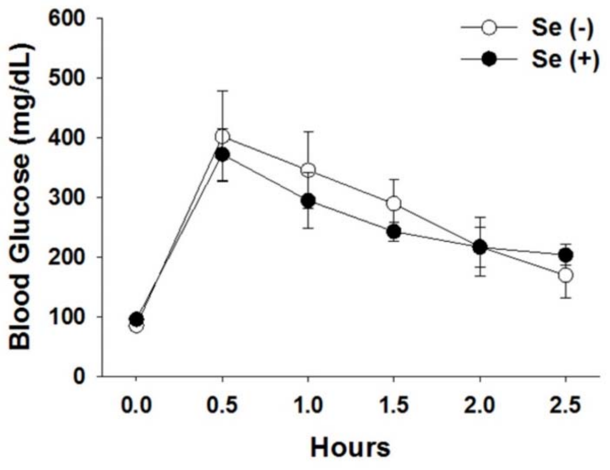

Figure S2. Glucose tolerance test in male *Terc*$^{+/+}$ mice at 12 months of age. Mice were fed a Se- or a Se+ diet since weanling. Levels of blood glucose were measured 0.5-2.5 hours after an intraperitoneal injection of glucose (1 g/kg body weight; n = 4). Se(-), selenium-deficient; Se(+), selenium-adequate.

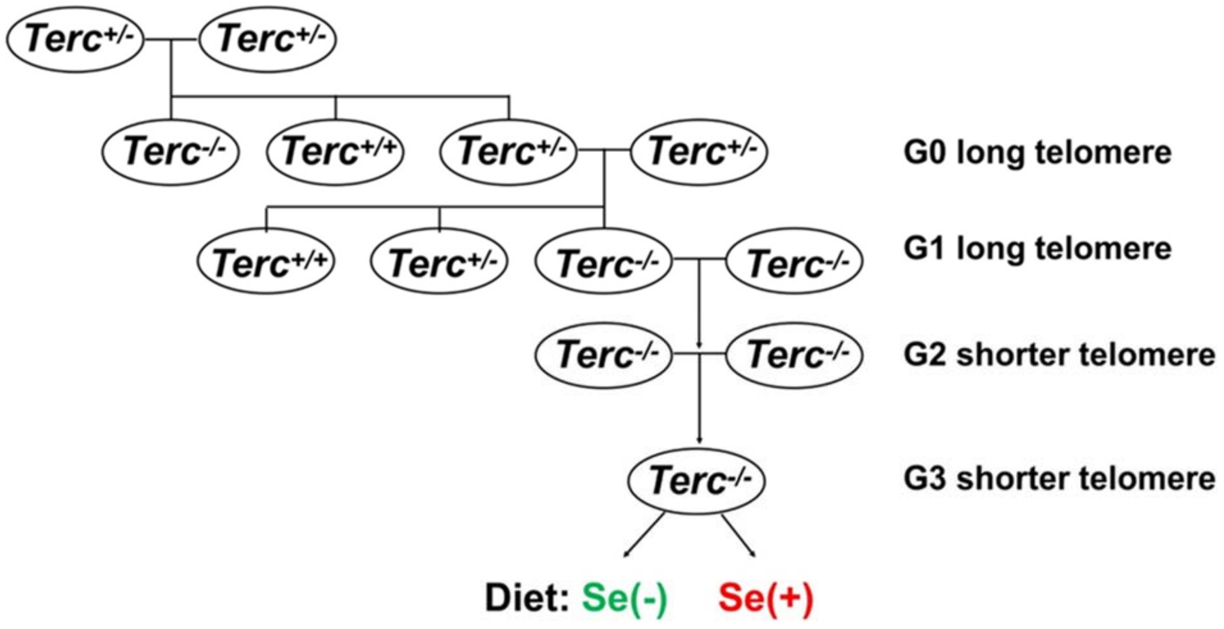

Figure S3. Breeding scheme for the generation of short telomere mice. G0-G3, generations 0-3. *Terc*$^{+/-}$ mice were interbred for 3-4 generations to generate sufficient quantity of the breeding G0 *Terc*$^{+/-}$ mice, followed by breeding of the G1-G3 *Terc*$^{-/-}$ mice. G3 *Terc*$^{-/-}$ mice were fed a Se(-) or a Se(+) diet since weanling until they were sacrificed at 12-24 months of age or died naturally. Se(-), selenium-deficient; Se(+), selenium-adequate.

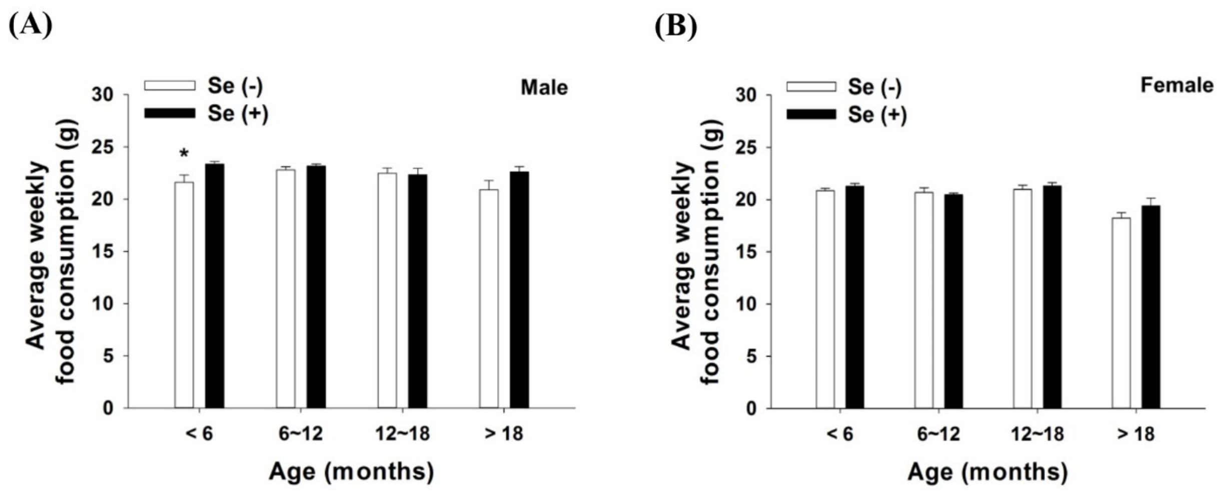

Figure S4. Average food intake in G3 *Terc*$^{-/-}$ mice. Male Se(-) *Terc*$^{-/-}$ mice consumed slightly, yet significantly less foods that those on a Se(+) diet at < 6 months of age. *, $P < 0.05$, compared to Se(-) mice. Se(-), selenium-deficient; Se(+), selenium-adequate.

(A)

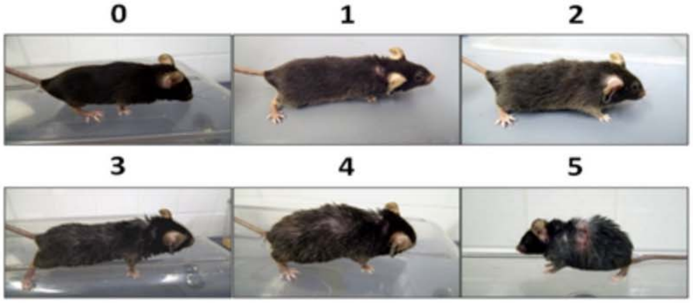

Male	Age (mo)	Score 5	4	3	2	1	0	n
Se (-)	7-9	1	2	24	5	0	0	32
	9-18	4	12	26	7	1	0	50
	> 18	2	8	9	1	0	0	20
Se (+)	7-9	0	1	7	20	3	0	31
	9-18	0	4	18	16	6	0	44
	> 18	0	3	10	3	1	0	17

(B)

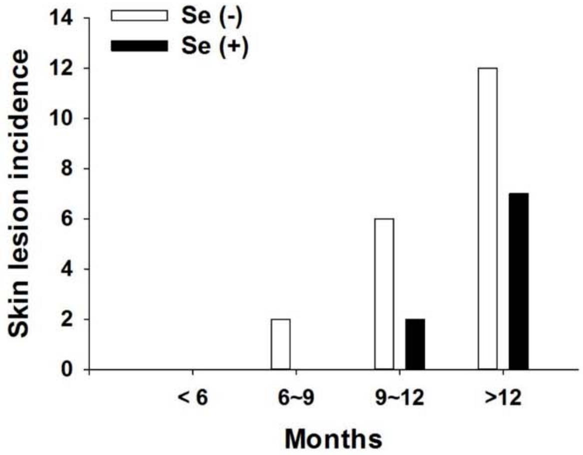

Figure S5. Skin aging in the male G3 *Terc*$^{-/-}$ mice. (A) References for hair graying and alopecia (0, normal; 5, most severe) and scores in adult Se- and Se+ G3 *Terc*$^{-/-}$ mice. (B) Time course of incidence of skin lesion in Se- and Se+ G3 *Terc*$^{-/-}$ mice. Mice were fed a Se- or a Se+ diet since weanling. The incidence of skin lesion is presented as the number of cases with open lesions being observed at the indicated age window. Se(-), selenium-deficient; Se(+), selenium-adequate.

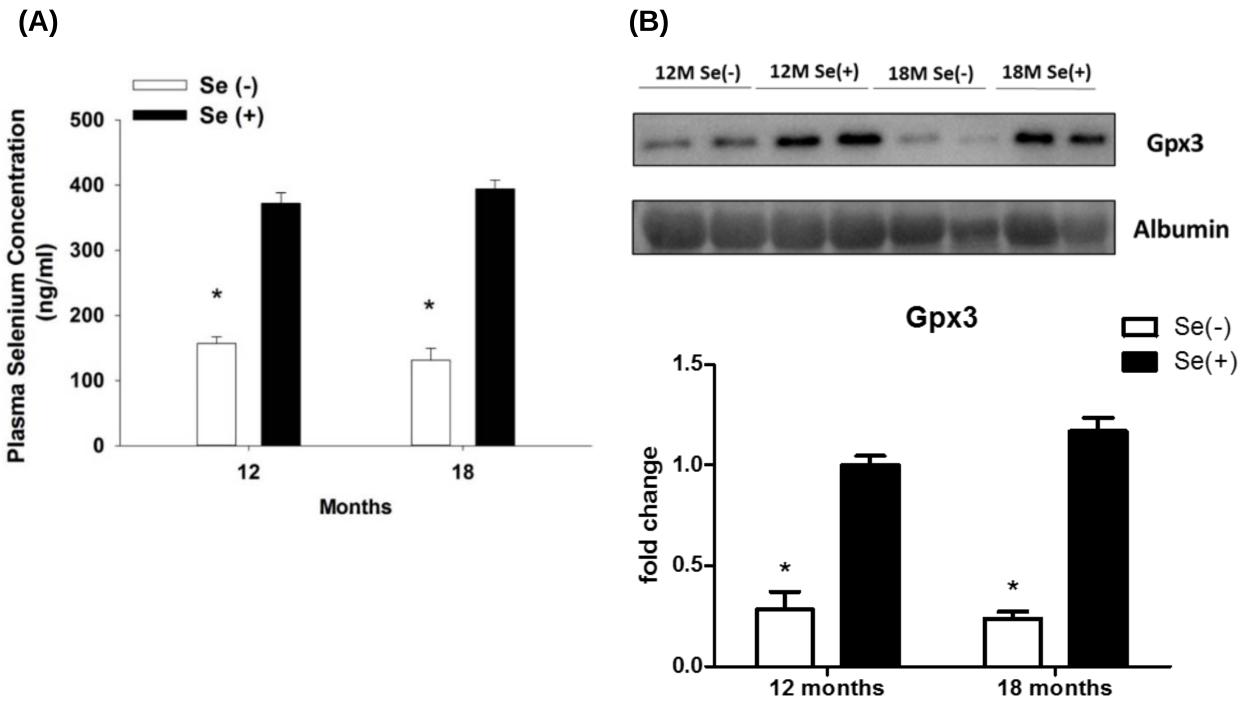

Figure S6. Se concentrations and glutathione peroxidase-3 protein levels in plasma of male G3 *Terc*$^{-/-}$ mice. Mice were fed a Se(-) or a Se(+) diet since weanling. (A) The Se concentrations were determined by hydride-generation atomic absorption spectrometry. *, $P < 0.05$, compared to Se(+) mice. (B) Western analyses and quantification of glutathione peroxidase-3 (Gpx3) protein levels in plasma (0.3 uL) of the mice. Values are means +/- SEM (n = 4). Se(-), selenium-deficient; Se(+), selenium-adequate.

(A)

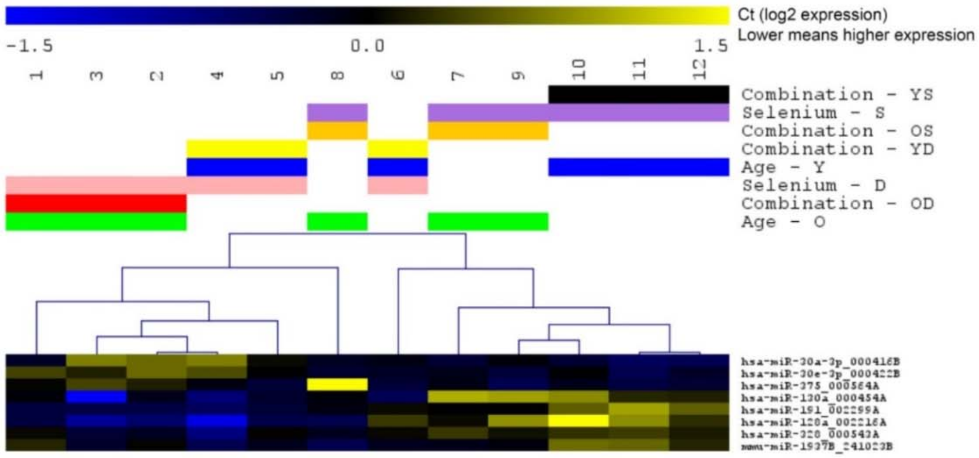
Ct (log2 expression)
Lower means higher expression
-1.5
0.0
1.5
1 3 2 4 5 8 6 7 9 10 11 12
Combination - YS
Selenium - S
Combination - OS
Combination - YD
Age - Y
Selenium - D
Combination - OD
Age - O
hsa-miR-30a-3p_000416B
hsa-miR-30e-3p_000422B
hsa-miR-375_000564A
hsa-miR-130a_000454A
hsa-miR-191_002299A
hsa-miR-128a_002216A
hsa-miR-328_000543A
mmu-miR-1937B_241023B

(B)

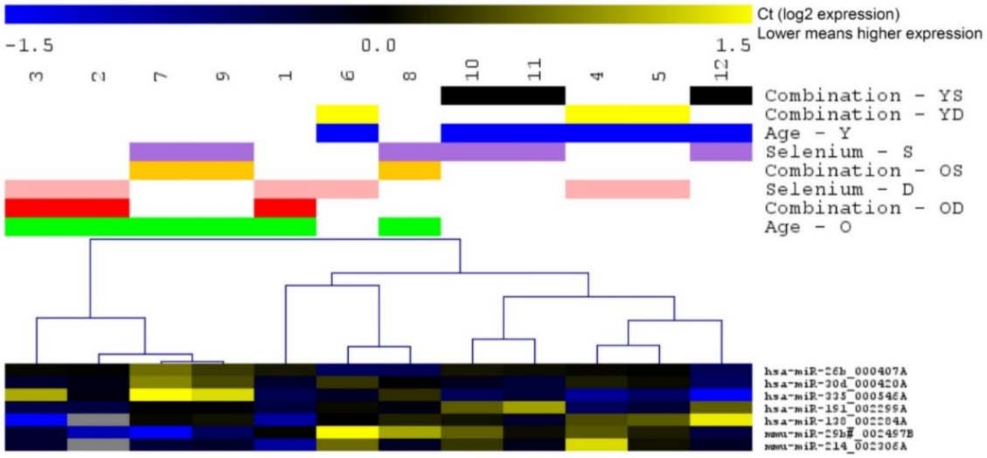
Ct (log2 expression)
Lower means higher expression
-1.5
0.0
1.5
3 2 7 9 1 6 8 10 11 4 5 12
Combination - YS
Combination - YD
Age - Y
Selenium - S
Combination - OS
Selenium - D
Combination - OD
Age - O
hsa-miR-26b_000407A
hsa-miR-30d_000420A
hsa-miR-335_000546A
hsa-miR-191_002299A
hsa-miR-138_002284A
mmu-miR-29b#_002497B
mmu-miR-214_002306A

(C)

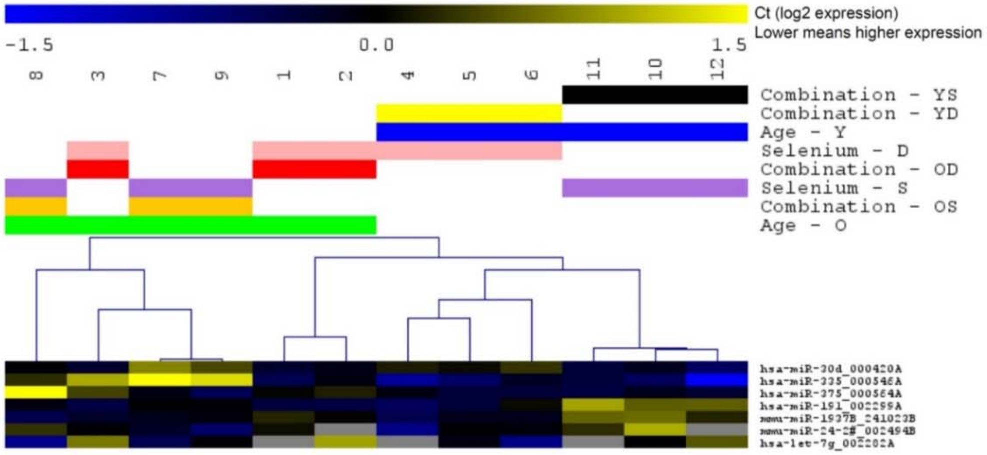
Ct (log2 expression)
Lower means higher expression
-1.5
0.0
1.5
8 3 7 9 1 2 4 5 6 11 10 12
Combination - YS
Combination - YD
Age - Y
Selenium - D
Combination - OD
Selenium - S
Combination - OS
Age - O
hsa-miR-30d_000420A
hsa-miR-335_000546A
hsa-miR-375_000564A
hsa-miR-191_002299A
mmu-miR-1937B_241023B
mmu-miR-24-2#_002494B
hsa-let-7g_002282A

(D)

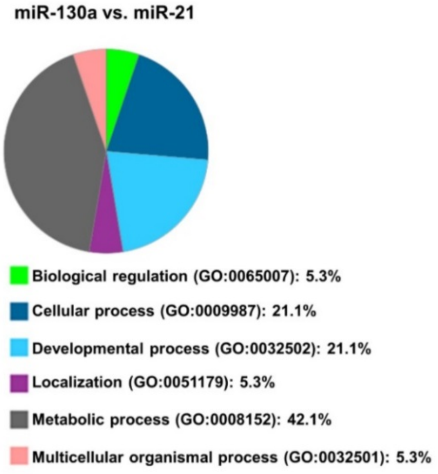

(E)

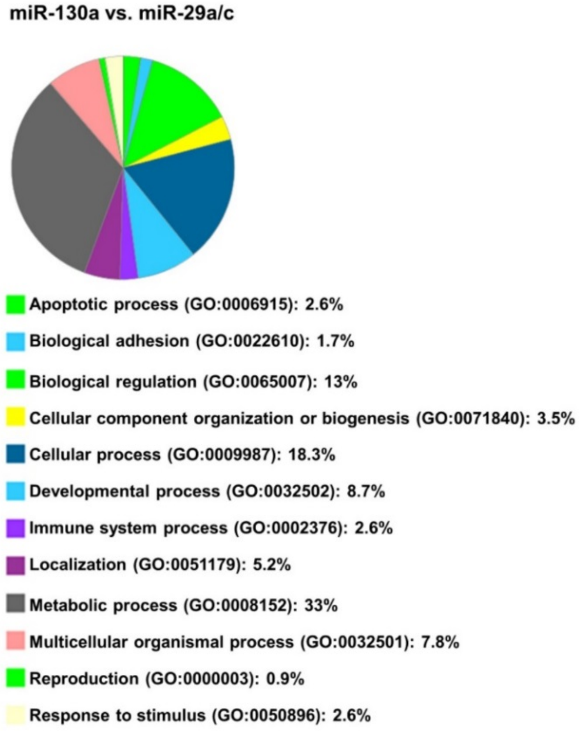

(F)

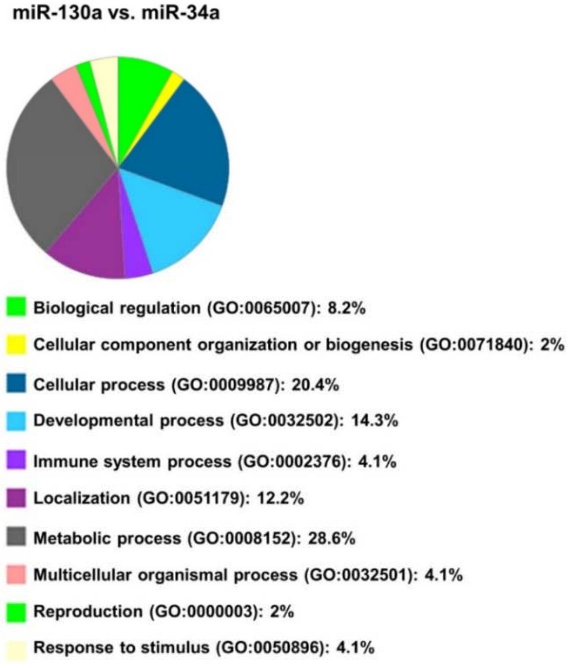

Figure S7. miRNA OpenArray analyses of plasma samples and the subsequent ontological analyses in male G3 *Terc*$^{-/-}$ mice at 12 and 18 months of age. Heat maps of differentially expressed miRNAs were shown as a result of dietary Se deprivation (**A**), aging (**B**), or both (**C**). miRNAs were detected using the Applied Biosystems TaqMan OpenArray system.

Each sample was analyzed using two ('A' and 'B') microfluidic cards, together featuring almost 650 murine miRNAs plus endogenous small RNA controls and negative controls. Reverse transcription and pre-amplification were performed with the manufacturer's reagents and per manufacturer's protocol (Applied Biosystems), with 14 pre-amplification cycles. RNA input was normalized by volume. Cards were loaded with pre-amplified cDNA mixed with polymerase master mix, and real time quantitative PCR was performed with an ABI 7900 real-time PCR machine at the Johns Hopkins University DNA Analysis Facility. Data were collected with the manufacturer's SDS software. RQ Manager software (Applied Biosystems) was used to process the array data. Thresholds, set at 0.2, were checked individually and corrected as necessary, and Cq values were extracted. Cq data were analyzed using the MultiExperiment Viewer (http://www.tm4.org/mev/). Heat maps were generated from median-centered data, analyzed by 2-way ANOVA for factors of selenium and age. Raw data have been deposited (GEO accession number: GSE71256). Following ontological analyses of TargetScan, results from Panther classification system indicated common pathways medicated by miR-130a and miR-21 (**D**), miR-130a and miR-29a/c (**E**), and miR-130a and miR-34a (**F**). Se(-), selenium-deficient; Se(+), selenium-adequate.

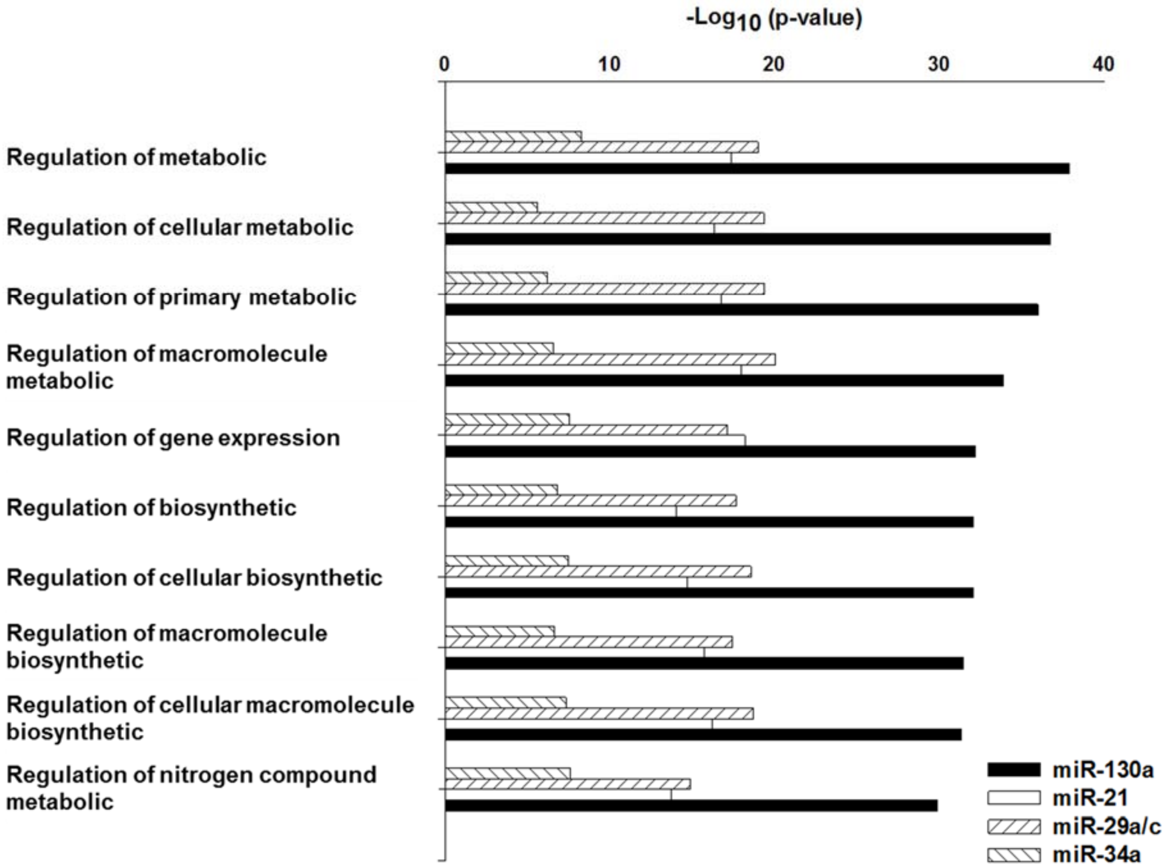

Figure S8. The top 10 biological pathways targeted by miR-130a, miR-21, miR-29a/c, and miR-34a. They were ranked by gene ontology enrichment analysis as detailed in "Gene Ontology Consortium: going forward. Nucleic Acids Res 43: D1049-1056, 2015".

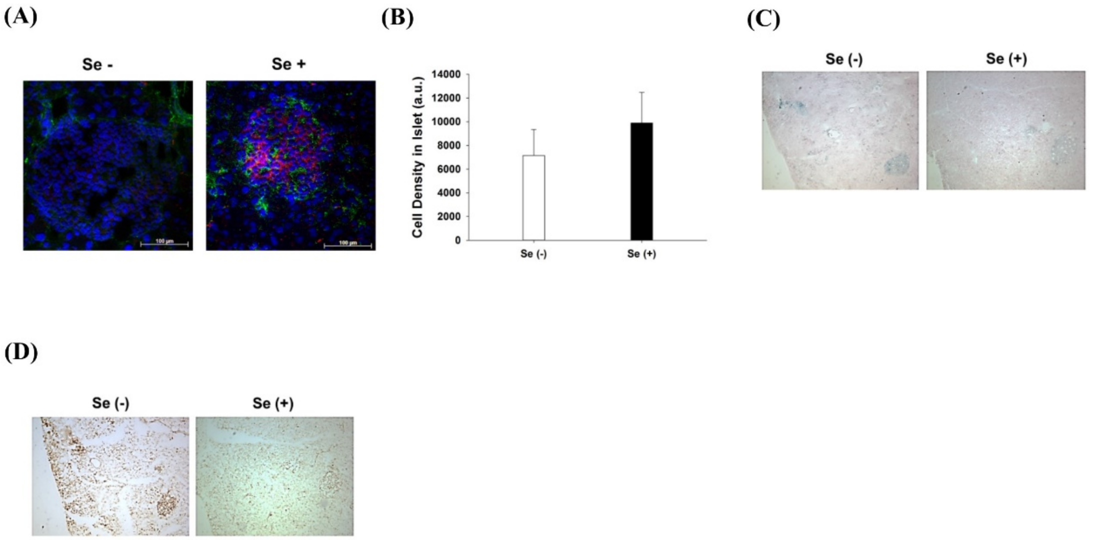

Figure S9. Representative pictures and quantification of cell density in pancreas of male G3 *Terc*$^{-/-}$ mice. (A) Representative images of immunohistochemistry of insulin and glucagon (40X; green, glucagon; red, insulin; blue, DAPI) in the pancreas. (B) Quantification of cell density in islets of the mice at 24 months of age. a.u., arbitrary unit. Representative pictures of senescence-associated β-galactosidase expression (C) and immunohistochemical analysis of γH2AX expression (D) in the pancreas. Se(-), selenium-deficient; Se(+), selenium-adequate.

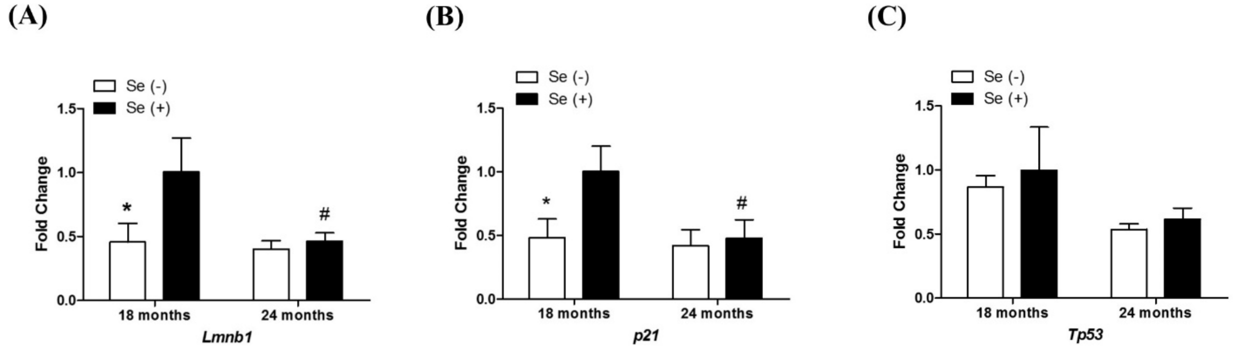

Figure S10. Effect of dietary Se deprivation and aging on mRNA expression of three senescence-related genes in pancreas of male G3 *Terc*$^{-/-}$ mice. Quantitative RT-PCR analysis of pancreatic *Lmnb1* (**A**), *p21* (**B**), and *Tp53* (C) mRNAs were performed. Values are means ± SEM (n = 6-9). *, $P < 0.05$, compared to Se+ mice; $^{\#}$, $P < 0.05$, compared to 18-month. Se(-), selenium-deficient; Se(+), selenium-adequate.

	12 month		18 month		
Parameter	Se (-)	Se (+)	Se (-)	Se (+)	
Triglycerides (mg dl-1)	44.6 (5.9) *	66.2 (6.0)	42.8 (3.3) *	74.2 (4.7)	Fasting
	49.4 (5.7) *	62.0 (4.3)	29.0 (1.3) * ‡	39.0 (3.3) ‡	Fed
Cholesterol (mg dl-1)	96.6 (2.6)	87.4 (7.2)	101.4 (6.8)	107.4 (9.7)	Fasting
	130.8 (5.9)	118.8 (7.9)	126.8 (3.0)	127.4 (8.5)	Fed
Glucose (mg dl-1)	167.4 (8.0) *	129.4 (13.2)	128.0 (11.1) * ‡	97.4 (5.2) ‡	Fasting
	192.6 (5.6)	196.8 (13.5)	175.2 (7.0)	206.2 (10.3)	Fed
Amylase (Ul-1)	2594.8 (173.0)	2614.6 (156.8)	2516.4 (249.4)	2351.6 (124.9)	Fasting
	3040.4 (175.9)	3136.0 (205.9)	2607.8 (132.9) ‡	2807.0 (102.1) ‡	Fed
Asp Aminotransferase (Ul-1)	59.6 (5.3) *	87.8 (14.4)	62.0 (2.8) *	124.8 (8.0) ‡	Fasting
	46.0 (3.0)	48.8 (4.3)	47.0 (2.5)	42.4 (1.6)	Fed
Ala Aminotransferase (Ul-1)	14.4 (2.0)	18.0 (4.5)	13.6 (2.1)	18.4 (2.9)	Fasting
	9.4 (2.3)	14.6 (5.3)	10.2 (1.3)	9.4 (2.5)	Fed
Creatine Phosphokinase (Ul-1)	126.0 (21.4)	68.2 (29.6)	96.4 (26.4)	131.4 (34.3)	Fasting
	113.6 (27.3)	67.0 (24.5)	57.8 (11.9)	70.4 (20.0)	Fed
Lactate Dehydrogenase (Ul-1)	243.6 (42.7) #	161.2 (8.4)	284.4 (24.1)	296.0 (33.1) ‡	Fasting
	194.0 (11.0)	190.2 (11.7)	207.4 (36.0)	159.4 (17.3)	Fed
Alkaline Phosphatase (Ul-1)	45.0 (5.0)	34.6 (4.0)	33.0 (3.4)	30.8 (3.5)	Fasting
	51.8 (3.3)	46.8 (4.4)	42.4 (1.8)	45.6 (2.3)	Fed
Bilirubin (mg dl-1)	0.58 (0.10)	0.54 (0.06)	0.48 (0.17)	0.28 (0.08)	Fasting
	0.16 (0.02)	0.24 (0.07)	0.14 (0.02)	0.26 (0.09)	Fed
Albumin (g dl-1)	3.72 (0.18)	3.56 (0.20)	3.08 (0.19) ‡	3.38 (0.15) ‡	Fasting
	4.02 (0.16)	3.58 (0.12)	3.54 (0.14)	3.60 (0.11)	Fed
Creatine (mg dl-1)	0.34 (0.02)	0.30 (0.03)	0.28 (0.02) ‡	0.24 (0.02) ‡	Fasting
	0.34 (0.02)	0.34 (0.02)	0.28 (0.02) ‡	0.30 (0.0) ‡	Fed
IGF-1 (ng ml-1)	331.5 (16.7) *	381.8 (30.4)	416.7 (31.7) ‡	349.9 (25.4)	Fasting
	518.9 (19.3)	537.6 (43.3)	538.7 (33.3) *	720.0 (42.6) ‡	Fed

Table S1. Biomarkers in plasma of Se-deficient and Se-adequate male G3 *Terc*$^{-/-}$ mice. *, *P* < 0.05, compared to Se(+); ‡, *P* < 0.05, compared to 12 months. #, *P* = 0.069, compared to Se(+). Se(-), selenium-deficient; Se(+), selenium-adequate.

Pathways	Definitions
Metabolic	The chemical reactions and pathways, including anabolism and catabolism, by which living organisms transform chemical substances. Metabolic processes typically transform small molecules, but also include macromolecular processes such as DNA repair and replication, and protein synthesis and degradation
Developmental	A biological process whose specific outcome is the progression of an integrated living unit: an anatomical structure (which may be a subcellular structure, cell, tissue, or organ), or organism over time from an initial condition to a later condition
Cellular	Any process that is carried out at the cellular level, but not necessarily restricted to a single cell. For example, cell communication occurs among more than one cell, but occurs at the cellular level

Table S2. Definition of the key pathways named in the Panther classification system. For details, see http://www.pantherdb.org/panther/category.do?categoryAcc=GO:0008152

Name	forward primer sequence	reverse primer sequence
β-Actin	CAGCCTTCCTTCTTGGGTATG	GGCATAGAGGTCTTTACGGATG
Ins1	AGAGAGGAGGTACTTTGGACTATAA	GCTTGCTGATGGTCTCTGATTA
Ins2	CCCTAAGTGATCCGCTACAATC	ACTCCCAGAGGAAGAGCA
Pdx1	CAGCCCTGAGCTTCTGAAA	GGAGCCCAGGTTGTCTAAAT
MafA	CAGCAGCGGCACATTCT	CCCGCCAACTTCTCGTATTT
Foxa2	GAGACTTTGGGAGAGCTTTGAG	GATCACTGTGGCCCATCTATTT
Tp53	CAGCTTTGAGGTTCGTGTTTG	AGTTCAGGGCAAAGGACTTC
P21	CCAGATAAGGTCGTGGTGAAA	GTGATGCTGACGGTGAACT
Lmnb1	CTCTCTCCAAGCCCTTCTTC	CTCCTCCACATCAACTCTCTTC

Table S3. Primers used for qRT-PCR analyses of pancreatic mRNA expression.

Parameters	Box #: Animal#: Body Weight:	Score	Date:	Date:	Date:	Date:	Date:	Date:
**Appearance**	General lack of grooming	1						
	Rough coat, ocular and nasal discharges	2						
	Piloerection, hunched up	3						
**Weight Loss**	Uncertain: body weight ↓< 10%	1						
	Intake: body weight ↓10~20%	2						
	No intake: body weight ↓> 20%	3						
**Skin Lesion (Size equals to the sum of the total skin lesion area)**	Skin lesion size as a dime (~18 mm)	1						
	Skin lesion size as a nickel (~21 mm)	2						
	Skin lesion size as a quarter (~25mm)	3						
**Natural Behavior**	Minor changes	1						
	Less mobile and alert, isolated	2						
	Vocalization, self-mutilation, restless or still	3						
**Dehydration**	Normal	1						
	Moderate	2						
	Severe	3						
**Provoked Behavior**	Minor depression or exaggerated response	1						
	Moderate change in expected behavior	2						
	Reacts violently, very weak, or in a pre-comatose state	3						
**Score**	If a score 3 was given more than once, score an extra point for each.	2-5						
	**Total**	0-20						

Table S4. The pain score sheet used in the study to monitor general health and behavior of the mice. This pain score sheet was developed together with, approved, and mandated by the IACUC of the University of Maryland. Scores 0-4, normal; 5-9, monitor carefully; 10-13, consider termination; >14, euthanization.
